# Metabolism and epigenetics in cancer: toward personalized treatment

**DOI:** 10.3389/fendo.2025.1530578

**Published:** 2025-07-25

**Authors:** Xiaoman Zhang, Dequan Liu, Sulan Yin, Yaru Gao, Xiaorui Li, Guangzhen Wu

**Affiliations:** ^1^ Department of Urology, The First Affiliated Hospital of Dalian Medical University, Dalian, China; ^2^ Department of Laboratory Medicine, The Faculty of Medicine and Pharmaceutical Sciences, Hainan Vocational University of Science and Technology, Haikou, China; ^3^ Department of Nursing, The Second Affiliated Hospital of Dalian Medical University, Dalian, China; ^4^ Department of Oncology, Cancer Hospital of Dalian University of Technology, Cancer Hospital of China Medical University, Liaoning Cancer Hospital and Institute, Shenyang, China

**Keywords:** cancer, glucose metabolism, lipid metabolism, epigenetics, metabolic reprogramming, tumor microenvironment

## Abstract

Epigenetic changes, such as DNA methylation, chromatin remodeling, and histone modifications, regulate gene expression without altering the DNA sequence. This review systematically analyzed over 500 studies including human cell line experiments (n>200), animal models (n>50), clinical cohort studies (n>100), and bioinformatics analyses retrieved from PubMed, Web of Science, and TCGA (The Cancer Genome Atlas). Studies increasingly show that genes involved in glucose and lipid metabolism, energy production, and modulation of metabolic hormones are regulated through epigenetic mechanisms. On the other hand, various metabolites participate in epigenetic modifications as coenzymes or substrates. Therefore, a greater understanding of the crosstalk between metabolism and epigenetics in cancer-related pathways could lead to the identification of key signaling molecules for targeted therapies, and raise the possibility of using dietary interventions to modulate epigenetic markers for individualized treatment. In this review, we have summarized the metabolic and epigenetic regulatory networks in cancer development, including glycolipid metabolic reprograming, the role of metabolites produced by the glut flora and tumor microenvironment, and key epigenetic drivers such as non-coding RNAs (ncRNAs). Data were curated from peer-reviewed articles, grounded in mechanistic studies using cell lines (SW480, MCF7 (Michigan cancer foundation-7)) and animal models (APC-mutant mice), with a focus on mechanistic studies, omics analyses, and translational research. Furthermore, we have discussed the potential of therapeutically targeting these pathways, along with the current challenges and future research directions, and a new strategy for reversing therapeutic drug resistance based on metabolism and epigenetic interaction was systematically explored.

## 1 Introduction

The occurrence and development of tumors are complex processes driven by multiple levels and dynamic molecular networks (1).With the in-depth study of epigenetic mechanisms, its central role in maintaining tumor cell identity, plasticity, and shaping intratumor heterogeneity has been revealed (2, 3). At the same time, the unique metabolic reprogramming characteristics of tumor cells, such as enhanced glycolysis, accelerated lipid synthesis and abnormal amino acid metabolism, have also had a profound impact on tumor biology on the premise of meeting the energy and biosynthetic requirements required for rapid tumor proliferation (4). These altered metabolic pathways produce a variety of specific metabolic intermediates and key substrates or cofactors that participate in and regulate epigenetic processes, such as histone modification and DNA methylation (5–7). The remodeling effect of metabolites on the epigenetic landscape plays a key connecting role in the cellular metabolic state and gene expression program (8, 9).

More critically, the interaction between metabolism and epigenetics is closely linked to the tumor microenvironment (TME) and immune regulation (10). On one hand, epigenetic modifiers regulate the expression of immune-related genes and influence the metabolic phenotype of tumor cells and the activity of immune cells (11). On the other hand, metabolites resulting from metabolic reprogramming in tumor cells, such as lactate and fatty acids, directly suppress the function of anti-tumor immune cells through epigenetic mechanisms, promote the activation of immune-suppressive cells, and facilitate tumor immune escape (12, 13). The gut microbiota, as an external driver, can promote tumor progression by regulating metabolites and immune responses when dysregulated. The interplay between metabolism, epigenetics, and immune regulation plays a key role in driving tumor progression (14). Our analysis integrated basic research (cell lines and animal models), clinical data (prospective cohorts), and omics datasets (TCGA, GEO (Gene Expression Omnibus)), including 387 cell line studies, 112 animal model investigations, and 45 clinical trials.

**Figure 1:**
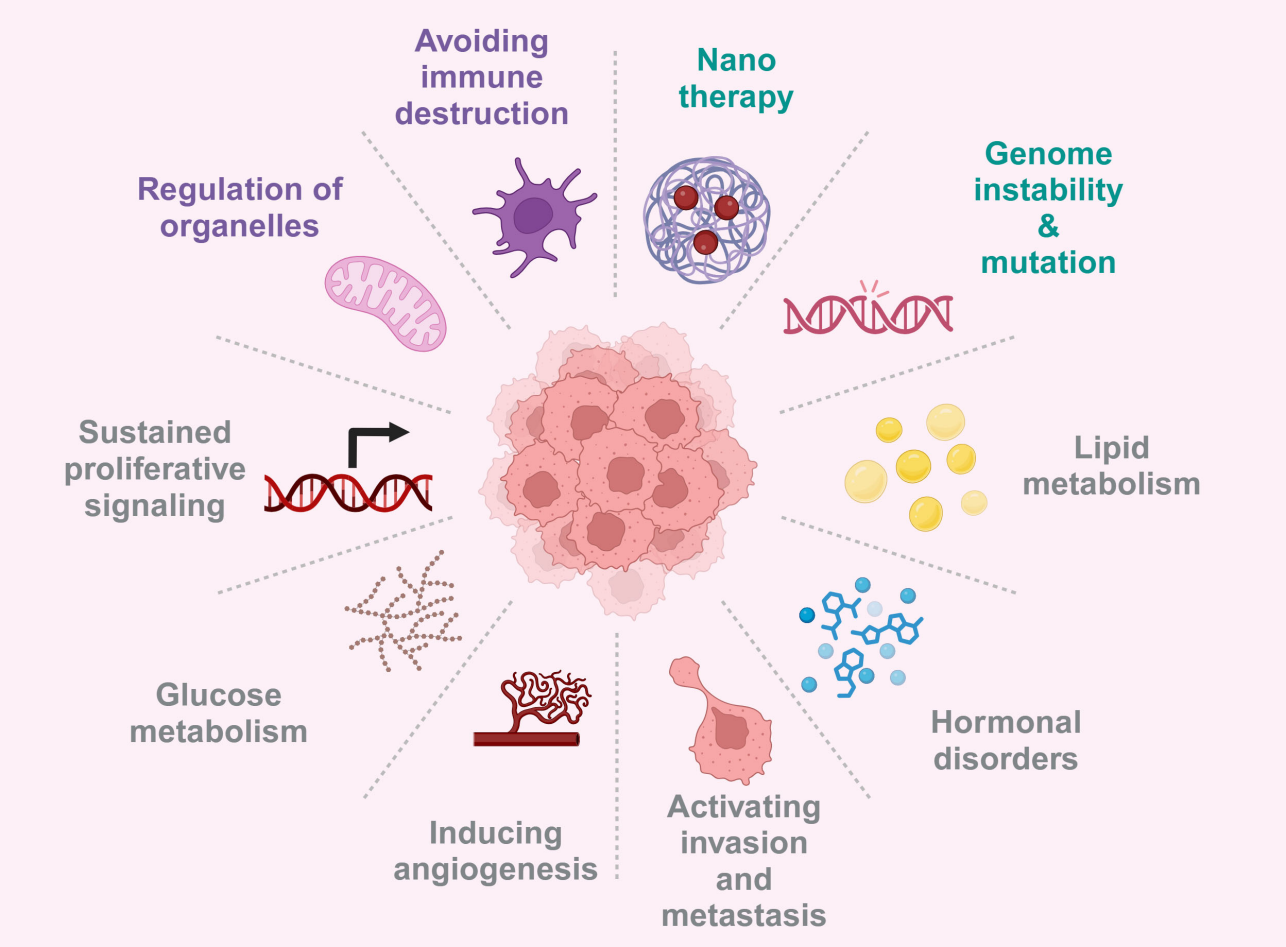
Tumorigenesis is driven by gene mutations, microenvironment abnormalities, metabolic reprograming, and hormonal dysregulation, thus provided multiple avenues for targeted therapies. Created with BioRender.com.

Metabolism and epigenetics and immune regulation play a key role in tumorigenesis, progression and treatment resistance (15, 16), the bidirectional regulatory mechanism between metabolism and epigenetics was verified in cell lines (SETD2 knockdown renal cancer cells) and mouse models (Kras mutated pancreatic cancer mice), which enabled us to have a more comprehensive understanding of their complex interactions and regulatory networks. In this review, we clarify the complex bidirectional regulatory relationship between metabolic reprogramming and epigenetic modification in tumors (17), how metabolites play the key role of regulatory factors in epigenetics, and how this interaction jointly promotes the progression of malignant tumors by influencing the immune microenvironment and non-metabolic pathways (18). We also explored targeted metabolic enzymes and epigenetic modification factors as key factors for developing new therapeutic directions, and proposed new insights for reversing treatment resistance and developing more precise anti-cancer therapies (19, 20) (**Figure 1**).

## 2 The modern era of epigenetic research

The study of whole-genome chromatin maps ushered in the era of modern epigenetic research (21). Epigenetics refers to inheritable changes in gene expression and cell phenotypes without altering the DNA sequence (**Table 1**).

**Table 1:** Epigenetic basis of molecular regulatory mechanisms and cellular processes.

Trait	Regulation mechanism	Reference
Reversibility	Methylated DNA can recruit MBDs, which attract other chromatin remodeling proteins that modify histones, such as HDAC, to form compact, inactive heterochromatin structures that inhibit gene expression.	(22)
Cellular memory	Antiviral memory B cells can be used for adaptive immune memory and innate immune memory at the same time to find targeted therapeutic drugs.	(23)
Stability	The fully reserved distribution of histone H3-H4 tetramer in cell division plays an important role in maintaining the epigenetic memory of cells.	(24)
Dynamic nature	AR binding induces an increase in FOXA1 and H3K27ac signaling, followed by increased chromatin accessibility, which is dynamic and closely related to gene expression regulation.	(25)
Interactivity	DNA methylation can affect the binding and activity of histone-modifying enzymes, while histone modification can also affect the recruitment and activity of DNA methylase.	(26)

AR, Androgen receptor; HDAC, Histone deacetylases; MBDs, Methyl-CPG binding domain proteins.

## 3 Metabolic reprogramming features

Metabolic reprogramming is a phenomenon wherein cancer cells adjust their metabolic pathways to adapt to the TME and higher energy needs (**Table 2**).

**Table 2:** Molecular basis of metabolic reprogramming in cancer cells.

Molecular metabolism level	Metabolite	Feature	Reference
Increased glycolysis	ATP	Metabolic intermediates supply the pentose PPP to promote macromolecular biosynthesis necessary for cancer cell growth and proliferation. Immune escape may be promoted by regulating the expression of PD-L1 on the surface of tumor cells.	(27)
Increased lipid metabolism	Acetoacetic acid, beta-hydroxybutyric acid and acetone	Altering the structure of its cell membrane, disrupting the environment in which molecularly targeted drugs act on the membrane, and interfering with the stability of the targeted drugs.	(28)
Changes in amino acid metabolism	Cystine transporter and BCAT1	Potential therapeutic targets for cystine transporters and BCAT1 to inhibit tumor growth and progression by inhibiting these targets.	(29)

PD-L1, Programmed Death-Ligand 1; BCAT1, Branched-Chain Amino Acid Transaminase 1; PPP, Pentose phosphate pathway.

## 4 Regulation of gene expression by epigenetic modifying enzymes

### 4.1 The central role of SETD2-mediated H3K36me3 modification in tumor suppression and immune regulation

Trimethylation of histone H3 lysine at position 36 (H3K36me3) is an epigenetic modification that regulates gene transcription and messenger RNA (mRNA) splicing. Histone methyltransferase SET domain containing protein 2 (SETD2), the key enzyme catalyzing this modification, has been identified as a tumor suppressor and immune modulator. For example, in terms of immune regulation, SETD2’s regulation of regulatory T cells (Tregs) affects tumor control and antiviral response (30, 31). In addition, SETD2 can enhance the expression/function of GATA binding protein 3 (GATA3) in intestinal derived thymic regulatory T cells (tTreg cells), thereby promoting the expression of ST2 (interleukin-1 receptor like 1, IL1RL1) (32). The absence of SETD2 in prostate cancer (PCa) cells promotes excessive activation of enhancer-binding protein 2 (EZH2), leading to an increase in whole genome H3K27me3 and chromatin repression, which in turn inhibits expression of tumor suppressor genes and promotes metastasis (33). Furthermore, the SETD2-knockout polycystic kidney disease-clear cell renal cell carcinoma (PKD-ccRCC) mouse model exhibits increased tumorigenesis and poor survival (34). This phenomenon may be partly attributed to the impaired heme synthesis and accumulated ferroptosis-related factors, which collectively create a pro-TME conducive to malignant progression (35). In addition to renal cancer, in pancreatic cancer cells, the deletion of SETD2 will promote the reorganization of acinar to ductal metaplasia (ADM) of pancreatic acinar cells driven by the oncogene KRAS, which is mainly mediated by F-box and WD repeat domain protein 7 (Fbxw7) (36). In the SGC-7901 human gastric cancer cells, restoration of forkhead box O transcription factors (FOXO) signaling pathway agonist or S IRT1 expression reverses the increase in proliferation and migration caused by SETD2 deletion (37). Smad7 is a negative feedback regulator of the TGF-β/Smad signaling pathway, and SETD2 deficiency leads to Smad7 down-regulation, which promotes TGF-β/Smad hyperactivation and the trans differentiation of myofibroblasts (38). In addition, SETD2 deletion can promote renal fibrosis by activating the TGF-β/Smad signaling pathway, even in the absence of Von Hippel-Lindau (VHL) protein (39). Thus, histone methyltransferases have an important role in tumorigenesis and cancer progression.

### 4.2 The synergistic regulation of m6A modification and histone demethylation drives tumorigenesis and drug resistance

**Figure 2:**
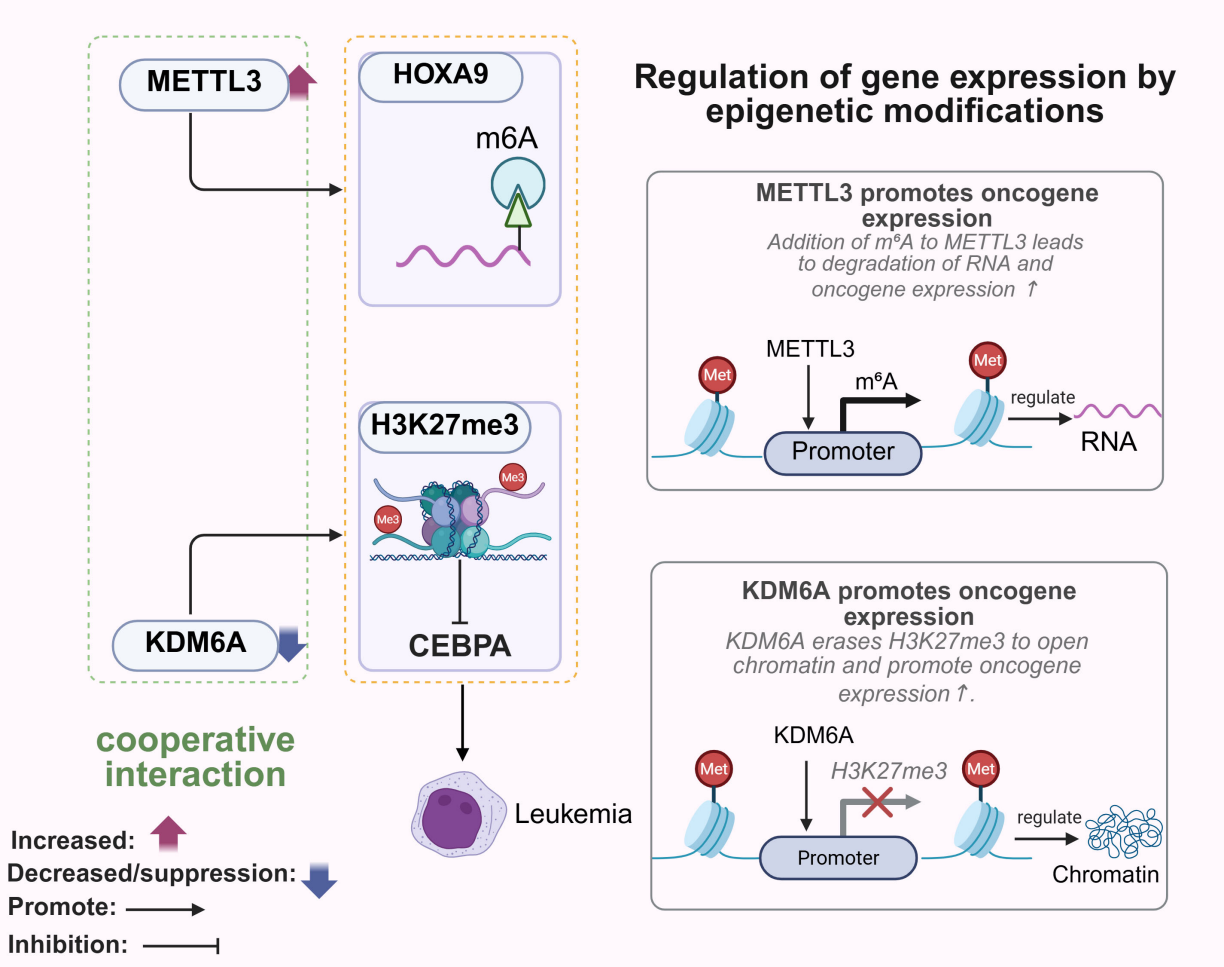
Role of methyltransferases and demethylases in tumorigenesis. METTL3 promotes m6A modification of HOXA9, whereas KDM6A deficiency leads to oncogene silencing by H3K27me3, which synergistically drives leukemogenesis. METTL3 affects RNA fate at the post-transcriptional level, and KDM6A determines the accessibility of gene transcripts through chromatin remodeling. METTL3, Methyltransferase-like 3, HOXA9, Homeobox A9. Created with BioRender.com.

N6-methyladenosine (m6A) is the most abundant RNA modification and plays a key role in transcriptional regulation (40). Methyltransferase-like 3 (METTL3) is central to the formation of m6A, which subsequently binds to specific RNA-binding proteins that influence metabolic processes (41). The combination of a DNA hypomethylating agent (HMA) with a PARPi showed potent anti-tumor effects against SETD2-deficient RCC cells (42). In breast cancer cells (BRCA), METTL3 can stabilize and upregulate the PD-L1 mRNA upon binding to m6A-modified IGF2BP3 (43). METTL3-mediated m6A modification of uncapped mRNA2235 (DCP2) triggers its degradation, and promotes mitosis and chemoresistance in small cell lung cancer (SCLC) cells through the PINK1/Parkin pathway (44). Knockdown of METTL3 inhibited Pin1-induced clonal expansion of the breast cancer MCF7 cells, but promoted the growth of 4T1 tumors *in vivo* (45). While METTL3 regulates gene expression at the post-transcriptional level, histone lysine demethylases (KDMs) determine the accessibility of gene transcription through chromatin remodeling (46). For example, KDM6A regulates chromatin structure and DNA accessibility by removing methyl groups from H3K27me3, and removal of this histone repressor mark activates gene transcription (47). METTL3-mediated m6A modification of the HOXA9 oncogene promoter, and oncogene silencing by H3K27me3 in the absence of KDM6A have been shown to synergistically drive leukemogenesis (48). YTH domain family protein 2 (YTHDF2), a reading protein that recognizes m6A and promotes RNA degradation, promotes tumor growth by facilitating degradation of target mRNAs (49). The METTL3-YTHDF2 axis can accelerate colorectal carcinogenesis through epigenetic suppression of YPEL5 (50). Furthermore, the chromatin remains in the transcriptionally repressed state in the absence of KDM6A, which can complement the regulatory activity of METTL3/YTHDF2 (51). Overall, histone methyltransferases (HMTs) and KDMs mediate chromatin accessibility and gene transcriptional activity by dynamically regulating histone post-translational modifications (PTMS), such as H3K27me3, and their dysfunction can drive tumorigenesis. Therefore, these epigenetic pathways are promising targets for overcoming cancer heterogeneity and drug resistance (52, 53) (**Figure 2**).

## 5 Metabolites regulate epigenetic pathways as substrates and cofactors

Metabolites are important substrates and cofactors for epigenetic modifications, linking cellular metabolism with gene regulation. The metabolic phenotype of tumor cells exhibits heterogeneity, involving multiple metabolic regions. Its high metabolic demand requires H ^+^ to accumulate in cells, thereby forming an acidic and oxidative TME (54, 55). The metabolic state of a cell can influence gene expression patterns through epigenetic mechanisms (56, 57). The metabolites produced during metabolic reprogramming of tumor cells, such as acetyl-CoA, NAD+, SAM, and αKG, can specifically affect epigenetic pathways (58). Acetyl CoA is produced during the metabolism of sugars, lipids, and proteins, and is involved in energy production, biosynthesis, and epigenetic regulation (59). The chemical modification and structural remodeling of chromatin depend on metabolic cofactors, and metabolite availability is therefore a direct indicator of changes in the epigenome (60, 61). CoA provides acetyl groups, promotes H3K27ac acetylation, determines histone acetyl transferase activity, and influences the expression of metabolism-related genes (62). The fluctuation of acetyl CoA levels plays a crucial role in regulating lipid synthesis. This regulation mainly occurs through epigenetic mechanisms: acetyl CoA dependent histone acetylation alters chromatin accessibility and structure, changes the chromatin status of lipid metabolism related genes, and affects the activity of related enzymes. Therefore, these changes affect the lipid synthesis pathway, leading to cellular metabolic disorders and cancer occurrence (63, 64). Targeting metabolic epigenetic interactions offers therapeutic potential. For example, in the phosphatidylinositol signaling pathway, breviscapine prevents the progression of metabolic stress-induced nonalcoholic steatohepatitis (NASH) by directly inhibiting TAK1 signaling (65).

Metabolic regulation plays a crucial role in various cellular processes (66), and is often impaired during carcinogenesis (67). Changes in metabolite levels may also affect epigenetic modifications by regulating the activity of specific enzymes (68). The metabolites and enzymes produced during metabolic reprogramming of tumor cells activate or inhibit certain epigenetic changes (69–71), such as DNA methylation and histone modifications, thereby coordinating cellular activity with altered nutrient availability (69). For instance, the catalytic activity of N-alpha acetyltransferase 40 (NAA40), a histone acetyltransferase (HAT), depends on acetyl CoA. The latter is a direct source of acetyl groups, which are transferred to specific sites on histone H4 by NAA40 (72). Thus, the increased abundance of acetyl CoA in tumor cells with high glucose metabolism may drive aberrant NAA40-mediated histone acetylation, resulting in the activation of pro-oncogenes (63). In addition, environmental carcinogens can hijack these metabolic and epigenetic pathways to promote cancer. Benzo (a) pyrene (BAP) is a polycyclic aromatic hydrocarbon (PAH) and potent organic toxicant, which forms reactive epoxide metabolites through metabolic activation. These metabolites can react with DNA to form adducts, leading to the mutation of key tumor suppressor genes, such as p53, which is the key mechanism of BaP induced lung carcinogenesis (73). Importantly, BAP exposure can also cause epigenetic dysregulation. It is associated with genome-wide DNA hypomethylation and may be caused by DNA methylation or inhibition of HDAC activity. This hypomethylation, coupled with the finding that this inhibition reduces the activities of biotin dependent enzymes, such as biotinidase (BTD) and holocarboxylase synthetase (HCS), which are themselves regulated by other epigenetic mechanisms, represents another important way for BAP to promote cancer development (74). Taken together, the metabolic heterogeneity of tumor cells affects histone diversity and epigenetic regulation, and plays a significant role in tumor progression (**Table 3**).

**Table 3:** Epigenetic modification of specific metabolites is a key factor in the development of cancer.

Specific metabolites	Histone modification	Metabolites as key factors in tumor induction	Reference
Acetyl-coa	As A cofactor for HATs, acetyl-coa is used as a donor for the acetyl group, which is transferred to the ϵ-amino side chain of the histone lysine residue.	It promotes lipid synthesis in tumor cells.	(75)
NAD+	NAD+ acts as a substrate to remove acetyl groups from histone tails, thereby altering chromatin structure and gene expression.	NAD+ can significantly enhance the sensitivity of anti-PD-1/PD-L1 antibody treatment.	(76, 77)
SAM	The transfer of methyl groups to specific amino acid residues of histones, forming methylation modifications, can affect chromatin structure and gene expression.	Tumor cells take up methionine from the environment via SAM, leading to a lack of SAM utilization in T cells and promoting tumor immune escape.	(78)
α-KG	Demethylases use α-KG as an auxiliary substrate to remove methylation-modifying groups from histone lysine residues by oxidative decarboxylation.	It is beneficial to maintain the fate of precancerous cells to promote the changes of chromatin and gene expression	(79)

NAD+, Nicotinamide adenine dinucleotide; SAM, S-adenosylmethionine; HATs, Histone Acetyltransferases; α-KG, Alpha-ketoglutaric acid.

### 5.1 Regulation of epigenetics by lipid metabolism

Increased *de novo* lipid synthesis is a key feature of many cancers (80). Cholesterol, an important component of the cell membrane, plays an indispensable role in cell growth and signaling (81, 82). Cholesterol, an important component of the cell membrane, plays an indispensable role in cell growth and signaling. Epigenetic mechanisms such as DNA methylation and histone modifications can regulate intracellular lipid concentration and signaling pathways (83). Recent studies have shown that the transcription factor Ikaros influences tumor development by modulating cholesterol metabolism pathways in the TME (84). Similarly, KLF10 exerts a protective effect in metabolic liver disease by regulating HNF4α-mediated metabolic pathways (85). Studies show that low-density lipoprotein (LDL) downregulates Krüppel-like factor 2 (KLF2) in endothelial cells through DNA and histone methylation, resulting in endothelial dysfunction and a hypercoagulable state (86). Furthermore, hyperlipidemia-induced coronary heart disease and peripheral artery disease have also been linked to epigenetic changes induced by circular RNAs (such as MICRA), and other RNA-level epitranscriptomics (87). Metabolite mediated epigenetic reprogramming further demonstrates this interaction. These findings collectively suggest that lipid metabolites are key epigenetic regulators. Among them, cholesterol biosynthetic intermediates, lipoproteins and acetyl CoA directly remodel chromatin structure through methylation, acetylation and RNA mediated mechanisms, thus linking metabolic disorders with tumorigenesis, vascular pathology and metabolic organ dysfunction.

### 5.2 Remodeling of gene expression by glucose metabolism

**Figure 3:**
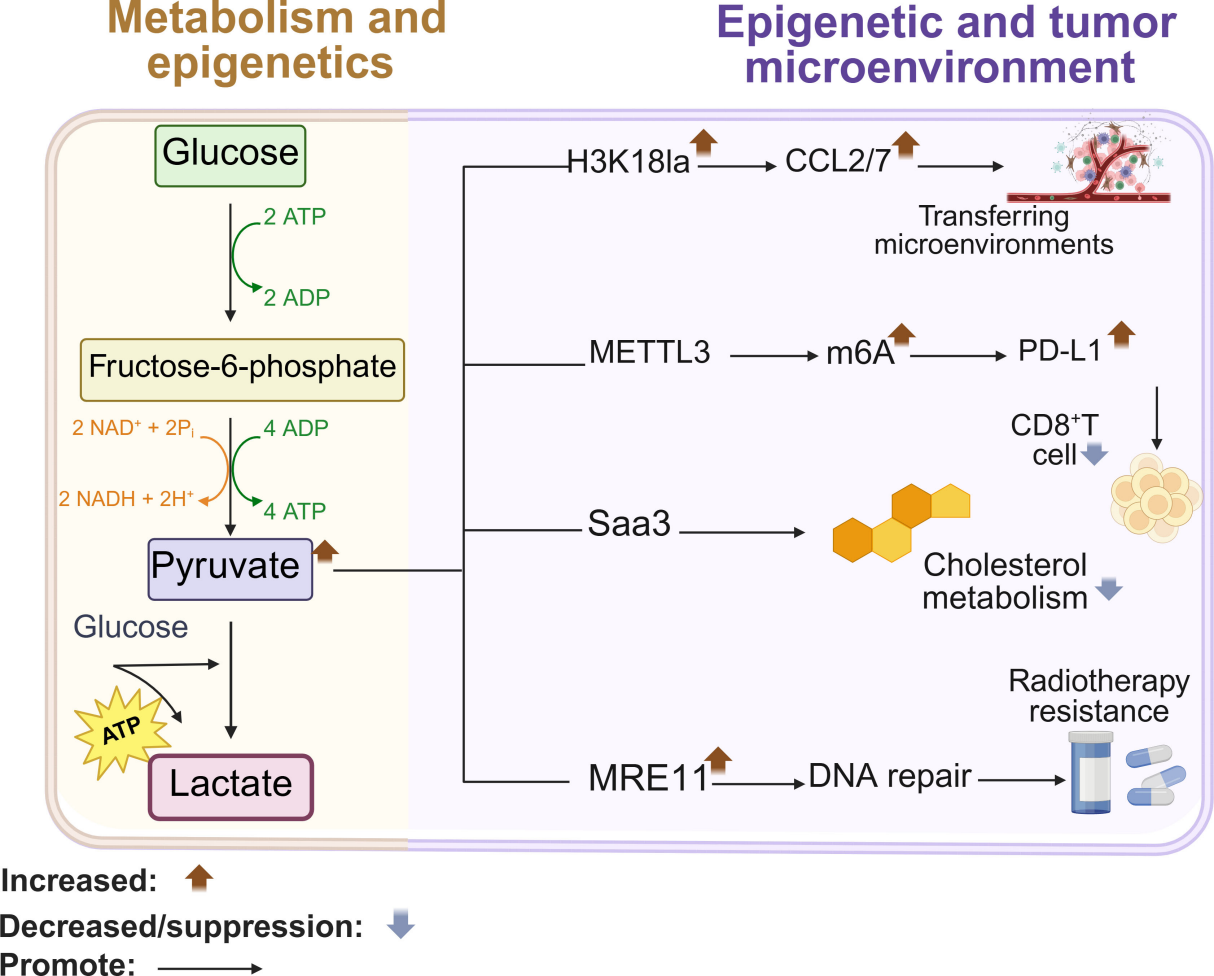
Glucose metabolism remodels gene expression. High glycolysis enhances transcription of the oncogene CCL2/7 through H3K18la modification, and promotes metastasis. Lactylation of METTL3 increases m6A modification in the PD-L1 mRNA promoter, resulting in increased transcript stability that eventually activates the immune checkpoint against T cells. Lactylation of MRE11 also promotes DNA repair and chemoresistance. Created with BioRender.com.

Glycolysis provides energy for lactate dependent epigenetic reprogramming. Elevated glycolysis in tumors generates lactate, driving lactylation – a novel post-translational modification that directly activates gene transcription through chromatin remodeling (6, 88, 89). Increased production of lactic acid in the TME is known to induce immunosuppressive conditions, which can mitigate the response to immunotherapies. In tumor cells, elevated glycolysis drives cancer cell metastasis by activating oncogenes ccl2/7 through h3k18 lactylation (h3k18la). Furthermore, lactate regulates epigenetic modifications by altering the chromatin (90). Specifically, lactate enhances the recruitment of the key homologous recombination (HR) protein MRE11 to DNA damage sites, thereby promoting DNA end resection and HR repair (91). Considering tumor metabolism and tumor immunity, exploring targeted lactoacylation has great potential for the development of cancer treatment strategies (89, 92). For example, two lactose modification sites were found in the zinc finger domain sample mettl3. Emulsification driven mettl3 mediated RNA m6A modification plays an important role in promoting tumor infiltrating myeloid cells (TIM) (93). In conclusion, glucose metabolism derived lactate regulates gene expression by remodeling the expression network of Pro metastatic genes, immunosuppressive genes, and genes related to genome stability through h3k18la, mettl3-m6a modification, and Mre11 mediated DNA repair triple epigenetic mechanism (**Figure 3**).

### 5.3 Conduct a systematic assessment of the hypothesis that “cancer is essentially a metabolic disease”

The assumption that cancer is fundamentally a metabolic disease: the assumption that metabolic reprogramming precedes and drives malignant transformation, but this assumption is still controversial at present. Although metabolic alterations are an indisputable feature of cancer, they are primary compared to gene mutations and require rigorous assessment. Studies have shown that mitochondrial dysfunction is observed in more than 80% of tumors. In models such as Kras mutant pancreatic cancer, damage to oxidative phosphorylation (OXPHOS) precedes genomic instability (94). Tumor metabolites, such as 2-HG from IDH mutations, directly disrupt epigenetic mechanisms, inducing hypermethylation and silencing tumor suppressors before significant mutations accumulate (95). Metabolic disorders, through endoplasmic reticulum stress, increase the apoptosis of cancer cells, thereby promoting the progression of endometrial cancer and inducing tumor occurrence and progression (96). However, most of the studies have limitations. For example, there are doubts about causal timing: whether metabolic changes precede driver mutations (TP53, APC) still has inferential significance (97). In addition, the Warburg effect in the hypoxic microenvironment may be a survival adaptation rather than a carcinogenic source, which is worthy of in-depth exploration (98). Although the origin remains unclear, the interaction between targeted metabolism and epigenetics shows clinical prospects: metformin takes advantage of the metabolic vulnerability of breast cancer, and its efficacy may depend on the STK11 status (99). hypothesis that cancer is essentially a metabolic disease is supported by certain evidence and shows potential in clinical applications, the causal relationship between metabolic reprogramming and genetic mutations, as well as the exact role of metabolic alterations in tumorigenesis, still require more comprehensive and in-depth research to clarify its scientific validity and practical significance.

## 6 Non-metabolic epigenetic mechanism based on enzyme translocation

Various intermediate metabolites can alter chromatin structure and function through chemical PTMS (100). Studies show that tumor metabolites are heavily influenced by the microenvironment (101), and enzyme translocation from the nucleus and mitochondria may alter the metabolism of cancer cells and their interactions with stromal cells in the TME (102). Apart from mediating metabolic reactions, enzyme translocation also regulates epigenetic pathways through non-metabolic effects (103). Therefore, the role of tumor metabolites in different organelles has important implications for the interplay between metabolism and epigenetics.

### 6.1 Nonmetabolic functions of the endoplasmic reticulum: calcium signaling and cholesterol homeostasis regulate tumor progression

The endoplasmic reticulum is the site of protein translation, folding and processing, as well as lipid secretion (104). Aberrant lipid metabolism or dysregulated ion transport in the endoplasmic reticulum can trigger organelle stress and tumorigenesis (105). Endoplasmic reticulum transmembrane protein 147(TMEM147) promotes the proliferation and metastasis of tumor cells, endows them with resistance to iron-mediated cell death, and induces polarization of M2-type macrophages by disrupting cholesterol homeostasis and increasing 27HC secretion (106). In addition, calcium ion in endoplasmic reticulum regulates the pathway related to tumor cell growth and drug resistance (107). Sigma-1 receptor (Sig-1R) is a molecular chaperone protein located in the endoplasmic reticulum, which plays a key role in regulating the endoplasmic reticulum calcium channel, which controls the growth of tumor cells and drug resistance (108). Sig-1R is located in the endoplasmic reticulum mitochondria associated membrane (MAM) domain. By sensing the change of calcium ion (CA ² + concentration) in the endoplasmic reticulum cavity, Sig-1R regulates Ca ² + signal transmission between mitochondria and cells, thereby affecting cell survival (109).

### 6.2 Targeted mitochondrial-related epigenetic reprogramming: a hub for metabolic adaptation and therapeutic resistance

Increased glycolysis may be related to mitochondrial dysfunction and enzymatic changes in tumor cells (110, 111). The key enzymes and intermediates of the glycolytic pathway can be used by tumor cells to synthesize proteins and nucleic acids, or protect mitochondrial function (112). On the other hand, mitochondrial intermediates initiate epigenetic pathways in the nucleus, and the resulting epigenetic marks regulate the expression of mitochondrial proteins (113, 114), a key bidirectional regulatory circuit is formed between the nucleus and mitochondria. For example, in pancreatic cancer, epigenetic defects of tumor cells (such as abnormal h3k27ac modification caused by setd2 deletion) were found to be associated with specific metabolic phenotypes (115). More importantly, epigenetic disorders (including abnormal histone modification and DNA methylation changes) can significantly change the functional state of mitochondria (116–118). Therefore, the regulation of nuclear gene expression by epigenetic mechanism directly affects the level and activity of these mitochondrial proteins, and then regulates the level of mitochondrial metabolites needed to maintain cell function (119). For instance, METTL17 regulates mitochondrial function in colorectal cancer (CRC) cells through epigenetic regulation (120). Therefore, exploring the interaction between epigenetic mechanism and mitochondrial function, especially the regulation of epigenetic mechanism on mitochondria, provides an important way for developing new targeted therapy strategies.

## 7 Metabolic reprogramming, sugar, lipid metabolism, crosstalk between epigenetics three relations

**Figure 4:**
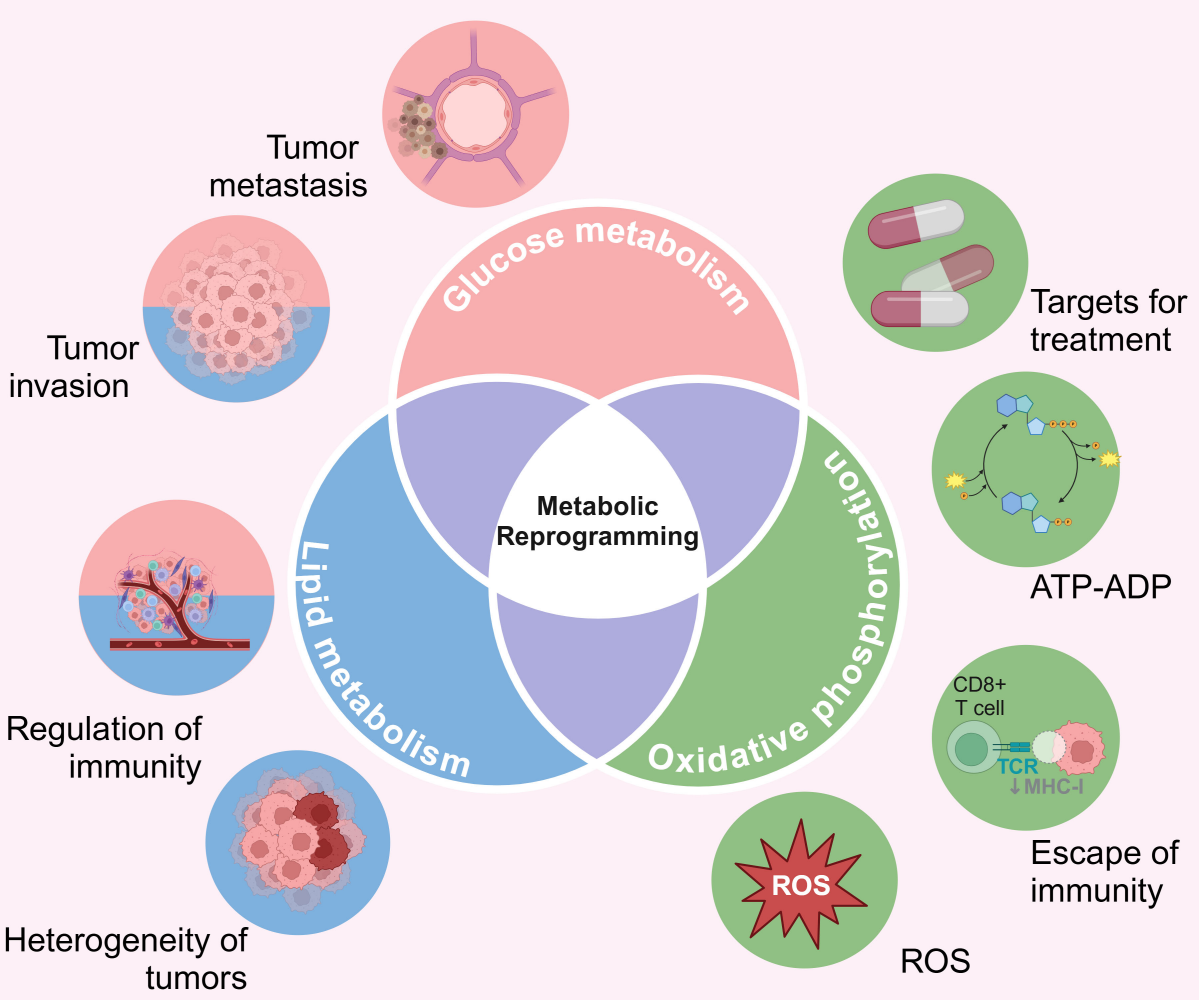
The cross-talk between metabolic reprogramming, glucose metabolism, lipid metabolism, and epigenetics. ATP, Adenosine triphosphate; ADP, Adenosine diphosphate; ROS, Reactive oxygen species. Created with BioRender.com.

Metabolic reprogramming can modulate the function of intra-tumoral immune cells by altering the concentration of intracellular metabolites (121–123), making it a key feature of tumorigenesis and progression. Tumor-derived exosomes (TDE) stimulate elevated nitric oxide synthase 2 (NOS2), thereby inhibiting mitochondrial oxidative phosphorylation, and promoting conversion of pyruvate to lactate (124). Furthermore, enhanced glycolysis in cancer cells increases acetyl Coa levels, which promotes up-regulation of oncogenes (MYC) through histone acetylation (125). Furthermore, epigenetic modifications can regulate the genes involved in glycolipid metabolism and promote tumorigenesis and progression (126). For example, increased methylation of the insulin gene (INS) promoter in pancreatic beta cells promotes gene silencing in diabetes (127). On the other hand, β-hydroxybutyric acid (ketone bodies) can activate antioxidant genes like FOXO3A by inhibiting HDACs and increasing histone acetylation (128). Therefore, the crosstalk between epigenetic mechanisms, glucose metabolism, and lipid metabolism regulates gene expression in metabolic diseases and cancer (**Figure 4**).

## 8 Internal and external factors jointly drive tumor progression through metabolism, immunity and epigenetics

### 8.1 External factors (microbiome) drive cancer by affecting internal metabolism and immunity

Clinical research on the effect of microbes on cancer began in 1868 and plays an important role in maintaining the ecological balance (129). The current scientific community believes that dysbiosis of gut microbiota is a hallmark of cancer (130, 131). The process of carcinogenesis is a highly complex and involves a variety of physiological and pathological events. Multiple sequencing methods have shown that the microbiota in the lung and intestine can cross-talk and are important components of TME (132). In CRC, which is the most common cancer, gut microbiota causes CRC by altering immune function (133, 134). Found microbes have the function of the dialectic, namely in the protection of human genes will damage to human multiple genes to form at the same time, its mechanism is mainly its ecological imbalance can by changing the host susceptibility to cancer events (including pathogenic microorganisms increase load) to significantly promote this process (135, 136). Gastrointestinal cancer is one of the main causes of cancer death at present. Intestinal microorganisms can damage cells or change the tumor immune microenvironment through direct or indirect effects to promote the development of gastrointestinal tumors (137, 138). Gut microbiota may be detecting high-risk PCa as a new useful marker, intestinal bacteria and their metabolites, short chain fatty acids (SCFAs), can promote PCa mouse model of cancer growth (139). Intestinal bacteria microbes in the estrogen group, namely, to metabolism of the aggregate of the intestinal bacterial genes of estrogen, increase the risk of BRCA in women (140). Symbiotic microbes are one of the factors influencing the antitumor immunity and treatment outcomes (141). The understanding of host-microbiome interactions and the assessment of microbial composition and function in patients provide a theoretical basis for subsequent targeted regulation and targeted interventions to reduce cancer risk (142, 143).

### 8.2 TME internal core process (metabolic reprogramming) drives cancer by regulating epigenetics and immunity

Metabolic rewiring of the tumor cells and immune cells regulates tumor progression by shaping the epigenome in TME (100). Although cancer is initiated by genomic alterations, it is more of a metabolic disease (144). Epigenetic pathways regulate gene expression levels by integrating environmental stimuli. Normal cells and cancer cells differ considerably in their metabolic levels (145, 146). Recent evidence suggests that a significant enhancement of the glycolytic pathway is a major feature of tumor cells (147), and energy production through glycolysis and lipid synthesis is fundamental to their ability to proliferate uncontrollably (148). Epigenetic regulation is highly sensitive to metabolic cues, and the metabolites in the TME can alter key epigenetic factors or enzyme activity to allow cancer cells to quickly adapt to the dynamic environment (149, 150). For instance, lactic acid and fatty acids can epigenetically regulate the function of immune cells and affect tumor resistance (151–153). Studies show that V-ATPase V0 subunit d2 (ATP6V0d2) expressed by macrophages inhibits tumor growth *in vivo*, while tumor-derived lactate inhibits ATP6V0d2 in macrophages, thereby promoting HIF-2α-mediated tumor progression (154). Lactate-derived histone lysine lactylation in the TME is a novel epigenetic modification that can directly stimulate chromatin structure and gene expression (6). TME reverses the effects of immune cells through a coordinated “regulatory triad”. Cancer cells compete with normal cells for key nutrients, which damage the immune cell function. The accumulation of tumor metabolites in the TME support the immunosuppressive cells and impair T cell function. At the same time, the spatial distribution, composition, and activation state of immune cells in tumor cells can influence the outcomes of immunotherapy (155, 156).

## 9 New insights into reversing treatment resistance and developing precise anti-cancer therapies

### 9.1 New insights into reversing resistance to therapy

The dysregulation of the interaction between metabolism and epigenetics can lead to drug resistance in tumor treatment. Recent studies have revealed innovative strategies targeting this intersection: First, overcome drug resistance through the combination of epigenetics and metabolic enzymes. For example, in renal cancer cells with SETD2 deficiency, the combination of DNA hypomethylation agents (HMA) and PARP inhibitors can overcome drug resistance by inducing synthetic lethal effects (42). This strategy is supported by preclinical data from 17 cell line studies and 3 phase II trials (NCT02850058, NCT03252097), as well as single-cell RNA-seq analysis of tumor-immune cell interactions. Second, intervention through the microbiome, metabolism and epigenetic axes. For example, animal models have confirmed that intragastric administration of butyrate can enhance the ability of chemotherapy drugs to penetrate the blood-brain barrier and reverse drug resistance in glioma. Personalized probiotic intervention based on microbiome characteristics is being verified for its sensitizing effect in clinical trials (157).

### 9.2 Developing precision cancer therapies using ncRNA delivery systems

Analysis of TCGA has revealed significant difference in the expression of ncRNAs between tumor tissues and normal tissues (158). NcRNAs are a group of heterogeneous transcripts that are not translated into proteins, but regulate gene expression at the post-transcriptional and post-translational levels (159–161). The m6A modification in the ncRNAs has been associated with gene expression levels and the biological behavior of tumor cells, thus providing potential new targets for cancer therapy (162, 163). For instance, several ncRNAs secreted by the Tumor-Associated Macrophage (TAMs) promote tumor proliferation, metastasis, angiogenesis, chemotherapy resistance, and immunosuppression (164, 165), and may also drive M1 or M2 polarization of the macrophages (166). By influencing molecular targets, ncRNAs influence alternative splicing (AS) processes and generate AS isomers, thereby promoting or inhibiting cancer signaling pathways (167). NcRNAs are also transported via extracellular vesicle (EVs) to regulate tumor development (168). For example, studies have confirmed that targeted delivery of miR-122 to liver cancer cells by cationic lipid nanoparticles (NPs) can inhibit angiogenesis and tumor growth (169). Similarly, the antitumor effects of siRNA and miRNA inhibitors have been verified in glioblastoma (170). In addition, the migration and clonal proliferation of A549 non-small cell lung cancer (NSCLC) cells were significantly inhibited by blocking the transcription factor -1(MALAT-1) mediated by RNA interference (171). In the liver metastasis model of colorectal cancer, the ncRNA delivery system has been precisely regulated. Specific delivery of miR-122 to hepatocytes through nanoparticles can down-regulate metastasis-related genes (MMPs), significantly inhibit tumor growth and prolong survival time, which provides a new idea for precise treatment (172). These innovative strategies based on the interaction between metabolism and epigenetics can reverse drug resistance, open up new ideas for precise anti-cancer treatment, and finally realize truly personalized cancer treatment.

## 10 Frontier progress and future directions of metabolism and epigenetic interaction in tumor research

At present, there is a lack of systematic analysis of intra-tumor heterogeneity. Future research should integrate single-cell multi-omics data (scRNA-seq, spatial metabonomics), use the database of Human Tumor Atlas Network and Tabula Sapiens, and combine the longitudinal clinical data of prospective cohort. The role of intestinal microflora needs to be verified by large-scale metabonomic research (American Intestinal Project and Human Microbial Project), and the relationship between microbial metabolites and epigenetic characteristics should be established (94). Key directions include: verifying the causal relationship between microbial metabolites (short-chain fatty acids) and epigenetic reprogramming in clinical cohort (137). At present, the treatment strategy for a single pathway faces challenges in terms of efficacy and specificity. Future efforts should focus on developing metabolically sensitive epigenetic regulatory factors as drug targets, for example, inhibiting tumor progression dependent on lipid synthesis by targeting acetyl-CoA-NAA40 axis (72). Additionally, exploring how non-coding RNAs regulate macrophage polarization by targeting metabolic-epigenetic crosstalk could further expand the repertoire of drug targets for remodeling the pro-TME (166). This aligns with the notion that innate immune cells, particularly macrophages, dynamically integrate metabolic cues and epigenetic reprogramming to shape the TME, as highlighted in the interplay between innate immunity and cancer pathophysiology (156). Specifically, tumor-derived lactic acid, as a key metabolic cue, modulates the activation and metabolic reprogramming of fibroblastic reticular cells in draining lymph nodes, thereby cooperating with innate immune cells to foster a pre-metastatic niche conducive to tumor progression (153). The integration of metabolism and epigenetic research represents the forefront of precision oncology. Future research must combine mechanism understanding with technological innovation to solve the problem of causality and transformation, so as to release the full potential of personalized cancer treatment.

## 11 Conclusion

In this review, we have explored the crosstalk between epigenetics and metabolism in tumor progression. While epigenetic mechanisms can affect metabolic reprogramming and immune infiltration in the TME, the local metabolites regulate tumor progression by targeting epigenetic factors. However, our knowledge of tumor metabolomics is incomplete, and the epigenetic mechanisms controlling glycolipid metabolism pathways, and their impact on tumor growth need to be explored. Advances in genomics and proteomics can provide new insights into metabolic mechanisms and key regulatory pathways, and help in the development of more effective therapies. A deeper understanding of the relationship between epigenetics and glycolipid metabolism in cancer will be of clinical significance, and pave the way for targeted and personalized therapies.
